# Depression, anxiety and coping strategies among Palestinian university students during political violence: a cross sectional study

**DOI:** 10.3389/fpubh.2024.1436672

**Published:** 2024-09-03

**Authors:** Muna Ahmead, Nuha El Sharif, Issa Abuiram, Eman Alshawish, Mohammad Dweib

**Affiliations:** ^1^Faculty of Public Health, Al Quds University, Jerusalem, Palestine; ^2^Faculty of Medicine and Health Sciences, Department of Nursing and Midwifery, An-Najah National University, Nablus, Palestine

**Keywords:** undergraduates, political violence, coping strategies, depression, anxiety

## Abstract

**Background:**

There are numerous wars and ongoing political violence in Palestine and little is known about how they have affected Palestinian undergraduate students’ mental health and coping strategies. This study aimed to assess the prevalence of depression, and anxiety symptoms and coping mechanisms among Palestinian university students during the times of current political violence in Palestine after October 7, 2023.

**Methods:**

A cross-sectional descriptive study design was utilized for a sample of students from 3 universities in Palestine (Al Quds University, Hebron University, and An-Najah University) and 1815 participants responded. Data were collected using self-reported questionnaires, including Hospital Anxiety and Depression Scale (HADS) and Brief COPE scale. Person correlation test, chi-square test, and bivariate analysis were performed to examine the associations between research variables.

**Results:**

The estimated prevalence of depression was 65.9, and 60.9% for anxiety. The logistic regression showed that students from the Arts Faculty, females, and those with working parents were significantly more likely to experience depression and anxiety symptoms. Also, active coping, emotional support, and humor reduced the likelihood of experiencing depression symptoms, while active coping, positive reframing, humor, and acceptance decreased the likelihood of developing anxiety symptoms. Further, the study found that using religion, self-blame, denial, and behavioral disengagement increased the likelihood of depression symptoms while planning, venting, religion, self-blame, denial, and behavioral disengagement increased the likelihood of anxiety symptoms.

**Conclusion:**

The study found that political violence often leads to symptoms of depression and anxiety among undergraduates. Furthermore, the use of maladaptive coping mechanisms increases the likelihood that these symptoms will occur. Providing immediate assistance to university students affected by political violence and conflicts is crucial for their emotional and mental recovery and coping with difficulties.

## 1 Background

The dangers of political violence and wars to national security have far-reaching consequences for people’s health, the economy, and overall quality of life (1). War or conflict is the deliberate use of military force or hostile activities by a government or organized non-state forces to cause harm to people and result in at least 25 fatalities (2). Most of these conflicts take place in Middle Eastern and African nations that have very low to moderate incomes, where health issues and economic status are closely related (3). More than half of the countries in the Eastern Mediterranean area are engaged in active armed conflict, affecting a total of 12 out of twenty-two countries (4–6). Major armed conflicts in recent times include conflict in Sudan, Gaza, and Ukraine (7). Research has investigated the influence of different wars, such as the Syrian conflict (8) or the conflict in Iraq (9), on particular populations. Both brief and long-term conflicts have detrimental effects on healthcare systems, displacing people from their homes and impeding their access to essential medical and mental services (4–6). By the end of 2022, over 60% or 25.8 million out of the total 43.3 million children who were forcibly relocated, experienced internal displacement due to violence and war (10). The investigation of the psychosocial well-being of people living in war-torn areas and areas affected by political violence has received considerable attention because of the high prevalence of mental health problems and increased vulnerability to signs of psychiatric disorders in the population (1). The widespread anxiety and uncertainty about the war are expected to have a long-term influence on the mental health of those affected by it (11). For instance, exposure to it can lead to mental health problems such as post-traumatic stress disorder, depression, anxiety, psychiatric morbidity, heightened risk of suicide, and emotional distress. Research has shown that there is a higher prevalence of depression (28.9%) anxiety (30.7%), and post-traumatic stress disorder (23.5%) among the population after experiencing political violence (12).

Furthermore, armed and violent conflicts detrimentally affect local universities and other educational institutions (13). This includes the shutdown of educational institutions, utilization of educational facilities by the military, and unfortunate fatalities and injuries among students and staff. Teachers’ absence may result from deliberate attacks, lack of security, and restricted mobility, which raises issues regarding the enrollment of students in universities (14). Moreover, university students may encounter many mental health problems that might affect their perspective, academic achievements, and capacity to adapt to daily routines (15). For instance, war and military conflicts have a strong link to anxiety and depression disorders. This is because they cause significant trauma to both mental and physical health (16). According to the American Psychiatric Association (2013), depression is defined as persistent feelings of sadness and hopelessness, accompanied by a decrease in motivation and interest in previously enjoyable activities (17). Furthermore, fear, whether triggered by physical dangers or subjective perceptions, becomes internalized and manifests as anxiety. Because of a depressive tendency, unconsciously anxious feelings can escalate into severe fear or panic (17). Research has shown that anxious and depressed students struggle with their academic pursuits and problem-solving abilities (18). Depression and anxiety are more common among university students than in the general population. Thabet et al. (19) conducted a study in which 399 university students from the Gaza Strip’s four main universities were selected randomly. According to the study, 10.3% of males and 13.8% of females reported feeling anxious. Furthermore, a study conducted in China by Yang and Yang (20) revealed that 44.64% of university students reported anxiety and 30.06% reported depression. Therefore, it is critical to consider these psychological effects on undergraduates in today’s world (21).

As a result of increased life-threatening anxiety and depression, students may feel a range of positive or negative emotions due to their inadequate coping strategies. Research suggests that problem-focused coping strategies are more beneficial for mental health, whereas emotion-focused approaches might have a negative effect (22, 23). War and political violence can have a major influence on people’s capacity to cope with stress, resulting in psychological problems such as depression, anxiety, substance abuse, aggressiveness, and addiction (24). On the other hand, prayer and family support are two coping mechanisms that might help survivors of political violence and conflict deal with their emotional distress and improve their mental health (22). Saxon et al. (25) found that the most commonly used coping mechanisms were acceptance, active coping, mental disengagement, planning, and instrumental and emotional social support. Gambling and drug use were uncommon. Academic institutions increasingly acknowledge the significance of enhancing the mental well-being of undergraduate students (26). Understanding students’ coping strategies directly or indirectly impacted by conflict is crucial for developing effective strategies to support their physical and psychological well-being (23).

In Palestine, the Israeli military has controlled the West Bank and Gaza Strip since 1967 (27). In 2023, the West Bank witnessed its worst year since 2005. Hundreds of Palestinians from the West Bank were killed. OCHA (28) and the Israeli military restricted movement between West Bank cities (29). Furthermore, the Gaza Strip has had four wars in the previous 13 years (2009, 2012, 2014, and 2021) (27). On October 7, 2023, Hamas conducted an attack that began a new conflict. Then Israel invaded and devastated Gaza. It is estimated that thousands of Palestinians have been killed or injured (28). The psychological and physical consequences of Israel’s border blockade and military occupation of Palestinians in the West Bank have been devastating (28). These consequences include a damaged economy, unemployment, altered living conditions, insufficient healthcare, and a general lack of optimism for the future. Currently, there is a lack of research that can provide stakeholders worldwide with information about the prevalence of anxiety and depression among undergraduate students, their coping strategies, and the factors that make them vulnerable during times of political violence and war in Palestine (21). To the best of the researcher’s knowledge, no previous research has looked at the frequency of anxiety and depression among West Bank undergraduates during periods of political violence and conflict. Given that young people will make up the bulk of the human workforce once they finish their university education, it is critical to emphasize their mental health, especially in the context of conflict. This study aimed to assess the prevalence of depression and anxiety symptoms, as well as coping strategies, among Palestinian university students in the West Bank during periods of current political violence and conflict following October 7, 2023. However, as the prolonged struggle between Israel and Palestine has persisted for over 75 years, the current study may assess the long-term effects of this conflict as well as the impact of the continuing conflict that began on October 7, 2023. It additionally sought to assess the association between depression and anxiety symptoms, as well as sociodemographic and psychological characteristics, and coping strategies. Finally, it aimed to investigate the factors that predict the development of depression and anxiety symptoms.

## 2 Materials and methods

### 2.1 Study design and sampling

This study aimed to assess the prevalence of depression and anxiety symptoms and coping strategies among Palestinian university students in the West Bank during the current political violence and war.

A descriptive cross-sectional study was carried out among undergraduate students at Al Quds University, Hebron University, and An-Najah University in West Bank. Al Quds University provides more than 120 undergraduate and postgraduate degrees. Its 15 faculties offer programs in medicine, life and natural sciences, business and management, arts and humanities, law and jurisprudence, engineering, and social sciences. Hebron University has 10 colleges: Islamic studies, Arts, Science, Technology, Agriculture, Medicine, Education, Finance, Management, Nursing, Pharmacy, and Graduate Studies. An-Najah University has 11 faculties spread across campuses, with 128 Bachelor’s degree programs, 78 Master’s and 13 PhD programs.

The study took place from December 7 to December 21, 2023, two months after the start of the Gaza War on October 7, 2023. These three universities are the largest Palestinian universities in the north, middle and south areas of Palestine. With a 0.05 significance level, 95% confidence level, and 0.05 accuracy, the study calculated a sample size of 1800 students. The participants were selected by using convenience sampling which is a non-probability technique used to select participants from the target population based on their easy accessibility (30). For data collection, an anonymous online self-administered survey method was used. As a result of the Israeli military’s blockade of Palestinian cities and restrictions on Palestinian travel in the West Bank, the researchers sent an electronic version of the questionnaire via Google Forms to the students at these three universities, along with an introductory invitation. The researchers sent the questionnaire online through student groups on Facebook, social media platforms, emails, and WhatsApp. A total of 1815 undergraduate students provided their responses.

### 2.2 Tool and measures

In this study, participants were asked to answer a self-reported questionnaire and it consisted of 3 sections.

Section one included a socio-demographic sheet to collect information related to the participant’s age, gender, faculty, income, place of residence, governorate of living (North governorate included Nablus, Jenin, Qalqilia, Tulkarim, Tubas and Salfeet cities, middle governorate included Ramallah, East Jerusalem and Jericho cities, and south governorate included Hebron and Bethlehem cities), marital status, faculty, father and mother education and work.

The second section had the Hospital Anxiety and Depression Scale (HADS) (31) which is a 14-item scale created to assess the presence of anxiety and depression. The HADS creates two scales to distinguish the two states: HADS–A for anxiety (seven questions) and HADS–D for depression (seven questions). On a 4-point severity scale, items are rated and each question is scored between 0 (no impairment) and 3 (severe impairment) with three denoting the highest anxiety or depression level. A case is considered conclusive if the score on either scale is greater than or equal to 11. A score of 0–7 is considered normal, 8–10 indicates mild depression, 11–14 indicates moderate depression and a score of 15–21 is equal to severe depression. The internal consistency coefficient (Cronbach’s *α*) was 0.825.

The third section had the Brief COPE scale that was developed by Carver in 1997 (32) and consisted of 28 questions. Both cognitive and behavioral strategies of coping are included and for each category, respondents indicate whether they have used a coping response on a four-point Likert scale (1 = I have not been doing this at all; 2 = I have been doing this a little bit; 3 = I have been doing this an average amount; 4 = I have been doing this a lot) and the higher score represents greater coping strategies used by the respondents. The Brief COPE scale assesses the following coping mechanisms: self-distraction, active coping, denial, substance use, emotional support, instrumental support, behavioral disengagement, venting, positive reframing, planning, humor, acceptance, religion, and self-blame. The internal consistency coefficient (Cronbach’s *α*) was 0.946.

A committee of five mental health experts reviewed the scale’s contents to ensure that the tool was culturally appropriate and no changes were made. The scale was first translated into Arabic by the research team and then back-translated to English by a licensed translator. At the pilot stage, we administered the tool to 20 undergraduate students to test for language clarity, both the original English questionnaire and the back-translated version were examined to ensure that the translation was accurate.

### 2.3 Data analysis

The data were analyzed with SPSS version 25 (IBM Corp., Chicago, IL, United States). The descriptive analysis for all study variables is reported as frequencies and percentages, means, and standard deviations. Pearson correlation test, chi-square test and T-tests were performed to investigate these associations. A multivariate regression analysis was carried out, and the results are presented as adjusted odd ratios (AOR) with a 95% confidence range. The adjusted model included all potential study confounders as well as factors associated with depression and anxiety that had a *p*-value of less than 0.05 in the bivariate analysis.

### 2.4 Ethical approval and consent to participate

All methods in this study were performed under the Declaration of Helsinki. The study was approved by the Al Quds University Research Ethical Committee (Ref No: 344/REC/2023). This online survey was anonymous. Written information about the aim of the study and how the data would be used was provided at the beginning of the study. Upon filling out the questionnaire, students provided informed consent for participation in this study.

## 3 Results

### 3.1 Socio-demographic characteristics of the participants

An online survey was completed by 1815 participants, who were on average 20.6 years old (SD 2.66, range 35 years), 40% were from Al Quds University and 29% were from Hebron University and An-Najah University. The bulk of participants (73.3%) came from faculties of health, and nearly one-third were third-year students. Additionally, 75.9% of the students were females, and nearly 70% of participants reported a family income of 1,600$ or less. Finally, 41.4 and 70.2% of the participants, respectively, stated that their mothers and fathers were unemployed during the war as seen in Table 1.

**Table 1:** Socio-demographic characteristics of participants.

Participants characteristics	*n*	%
University		
Al Quds University	762	42.0%
Hebron University	526	29.0%
An-Najah University	527	29.0%
Study year		
First-year	258	14.0%
Second-year	413	22.0%
Third-year	604	33.0%
Fourth-year	438	24.1%
Fifth and six-year	102	5.6%
Faculty		
Faculties of Science	212	11.7%
Faculties of Art (Humanities)	272	15.0%
Facilities of health	1,331	73.0%
Gender		
Male	437	24.0%
Female	1,378	75.9%
Place of living		
City	836	46.1%
Village	892	49.1%
Refugee camp	87	4.8%
Marital status		
Single	1,682	92.7%
Not single	133	7.3%
Family income ($)**		
Less than 500	229	12.6%
500 to 1,000	614	33.8%
1,001 to 1,600	418	23.0%
More than 1,600	395	21.8%
No income	159	8.8%
Area		
Southern area	817	45.0%
Middle area	498	27.4%
Northern area	500	27.6%
Father’s education		
≤12 years of schooling	962	53.0%
> 12 years of schooling	853	47.0%
Father’s work		
No work	752	41.4%
Currently working	1,063	58.6%
Mother’s education		
≤12 years of schooling	926	51.0%
> 12 years of schooling	889	49.0%
Mother’s work		
No work	1,274	70.2%
Has work currently	541	29.8%

*N = 1851 for all variables.*

*SD, standard deviation.

**$, American dollars.

### 3.2 The prevalence of depression and anxiety symptoms

The findings revealed that the overall mean for depression was 12.14 (SD 4.35) and for anxiety was 11.66 (SD 4.72). Also, the findings showed that (65.9%) of the sample met the clinical cut-off for depression (a score equal to or greater than 11), which means they are at high risk of meeting a clinical diagnosis of depression. Additionally, 60.9% of the sample met the clinical cut-off for anxiety, as shown in Table 2.

**Table 2:** Prevalence of depression and anxiety symptoms among study sample.

	Depression		Anxiety	
Scale cut-off point	*n*	%	*n*	%
<11	619	34.1%	710	39.1%
≥11	1,196	65.9%	1,105	60.9%

Total *N* = 1851.

### 3.3 The association between depression, anxiety, and sociodemographic variables

Chi-square test assessed the associations between depression symptoms and respondent characteristics. The results, as seen in Table 3, demonstrated significant relationships between depression and the university in which the student enrolled with Al Quds University having the highest percentage (38.5%, *p*-value = <0.001). Significant correlations were also found between depression and the following variables:, gender (female, 79.3%, *p*-value = <0.001) place of living (villages, 51.6%, *p*-value = 015), marital status (single, 91.8%, *p*-value =0.049), family income (group from 500 to 1,000$: 35.6%, *p*-value = 0.01), district (South district, 48.1%, *p*-value = <0.001), father education (≤12 years group, 55.2%, *p*-value =0.010) father work (has work currently, 54.6%, *p*-value = < 001), mother’s education (≤12 years group, 52.9%, *p*-value =0.024) and mother work (did not have work, 72.5%, *p*-value = 0.003).

**Table 3:** The bivariate correlations between depression and sociodemographic variables in the sample.

	Depression				
	<11		≥11		
	Total *N* = 619		Total *N* = 1,196		Chi-square
	*n*	%	*n*	%	*P*-Value
University					
Al Quds University	302	48.8%	460	38.5%	<0.001
Hebron University	149	24.1%	377	31.5%	
An- Najah University	168	27.1%	359	30.0%	
Study year					
First-year	100	16.2%	158	13.2%	0.127
Second-year	132	21.3%	281	23.5%	
Third-year	189	30.5%	415	34.7%	
Fourth-year	159	25.7%	279	23.3%	
Fifth and six-year	39	6.3%	63	5.3%	
Faculty					
Science faculties	69	11.1%	143	12.0%	0.373
Faculties of art (Humanities)	84	13.6%	188	15.7%	
Health sciences faculties	466	75.3%	865	72.3%	
Gender					
Male	189	30.5%	248	20.7%	<0.001
Female	430	69.5%	948	79.3%	
Living place					
City	311	50.2%	525	43.9%	0.015
Village	275	44.4%	617	51.6%	
Refugee camp	33	5.3%	54	4.5%	
Marital status					
Single	584	94.3%	1,098	91.8%	0.049
Not single	35	5.7%	98	8.2%	
Area					
Southern area	242	39.1%	575	48.1%	<0.001
Middle area	215	34.7%	283	23.7%	
Northern area	162	26.2%	338	28.3%	
Father’s education					
≤12 years of schooling	302	48.8%	660	55.2%	0.010
> 12 years of schooling	317	51.2%	536	44.8%	
Father’s work					
No work	209	33.8%	543	45.4%	<0.001
Currently working	410	66.2%	653	54.6%	
Mother’s education					
≤ 12 years of schooling	293	47.3%	633	52.9%	0.024
> 12 years of schooling	326	52.7%	563	47.1%	
Mother’s work					
No work	407	65.8%	867	72.5%	0.003
Has work currently	212	34.2%	329	27.5%	
Family monthly income ($)*					
Less than 500	59	9.5%	170	14.2%	<0.001
500 to 1,000	188	30.4%	426	35.6%	
1,001 to 1,600	158	25.5%	260	21.7%	
More than 1,600	160	25.8%	235	19.6%	

No significant difference was found by age (*t*-test *p*-value = 0.518).

*$, American dollars.

Additionally, chi-square test was used to assess the associations between anxiety symptoms and respondent characteristics. The results, as seen in Table 4, demonstrated a significant relationship between anxiety and the university in which the student enrolled, in with Al Quds University had the highest percentages (36.3%, *p*-value = <0001). Significant correlations were also found between anxiety and the following variables: gender (Female, 82.2%, *p*-value <0.001*), place of living (Villages, 54.4%, *p*-value = 0.006), family income (group from 500 to 1,000$, 36.8%, *p*-value = <0.001), district (south district, 47.9%, *p*-value = <0.001), father education (≤12 years, 54.9% *p*-value = 0.040), father work (has work currently, 53.9%, *p*-value = <0.001), mother’s education (≤12 years group, 53.3%, *p*-value =0.015) and mother work (did not have work, 73.3%, *p*-value = < 0.001).

**Table 4:** The bivariate correlations between anxiety and the study’s sociodemographic.

Sociodemographic variables	Anxiety				
	<11		≥11		
	Total *N* = 710		Total *N* = 1,105		Chi-Square
	*n*	%	*n*	%	*P*-value
University					
Al Quds University	361	50.8%	401	36.3%	<0.001
Hebron University	166	23.4%	360	32.6%	
An-Najah University	183	25.8%	344	31.1%	
Study year					
First-year	118	16.6%	140	12.7%	0.057
Second-year	163	23.0%	250	22.6%	
Third-year	212	29.9%	392	35.5%	
Fourth-year	175	24.6%	263	23.8%	
Fifth and six-years	42	5.9%	60	5.4%	0.409
Faculty					
Science Faculties	74	10.4%	138	12.5%	
Faculties of art (Humanities)	108	15.2%	164	14.8%	
Health sciences faculties	528	74.4%	803	72.7%	
Gender					
Male	240	33.8%	197	17.8%	<0.001
Female	470	66.2%	908	82.2%	
Living place					
City	342	48.2%	494	44.7%	0.351
Village	335	47.2%	557	50.4%	
Refugee camp	33	4.6%	54	4.9%	0.266
Marital status					
Single	664	93.5%	1,018	92.1%	
Not single	46	6.5%	87	7.9%	
Area of living					
Southern area	288	40.6%	529	47.9%	<0.001
Middle area	245	34.5%	253	22.9%	
Northern area	177	24.9%	323	29.2%	
Father’s education					
≤12 years of schooling	355	50.0%	607	54.9%	0.040
> 12 years of schooling	355	50.0%	498	45.1%	
Father’s work					
No work	243	34.2%	509	46.1%	<0.001
Has work currently	467	65.8%	596	53.9%	
Mother’s education					
≤12 years of schooling	337	47.5%	589	53.3%	0.015
> 12 years of schooling	373	52.5%	516	46.7%	
Mother’s work					
No work	464	65.4%	810	73.3%	<0.001
Has work currently	246	34.6%	295	26.7%	
Family monthly income ($)*					
Less than 500	67	9.4%	162	14.7%	<0.001
From 500 to 1,000	207	29.2%	407	36.8%	
1,001 to 1,600	189	26.6%	229	20.7%	
More than 1,600	191	26.9%	204	18.5%	
No income	56	7.9%	103	9.3%	

No significant difference was found by age (*t*-test *p*-value = 0.970).

*$, American dollars.

### 3.4 Associations between coping strategies, depression, and anxiety

Pearson correlation was used to investigate the associations between coping strategies and depression and anxiety symptoms as seen in Table 5. There were significant negative weak correlations between depression and active coping, emotional support, humor, acceptance, self-distraction, and substance use. Also, there were significantly positive weak correlations between depression and planning, venting, religion, self-blame, denial, and behavioral disengagement. For anxiety, there were significantly positive weak correlations between anxiety and active coping, use of informational support, positive reframing, planning, emotional support, venting, religion, self-blame, self-distraction, denial, and behavioral disengagement.

**Table 5:** The correlation between coping strategies, depression and anxiety.

		Depression		Anxiety	
	Coping Mean (SD)	Pearson correlation	*P*-value	Pearson correlation	*P*-value
Coping strategies					
Active coping	4.92 (±1.65)	−0.057^*^	0.015	0.062	0.008
Use of informational support	3.92 (±1.66)	0.018	0.433	0.151	<0.001
Positive reframing	4.39 (±1.84)	0.034	0.147	0.067	0.005
Planning	4.67 (±1.61)	0.185	<0.001	0.310	<0.001
Emotional support	3.79 (±1.63)	−0.004	0.858	0.120	<0.001
Venting	4.50 (±1.57)	0.149	<0.001	0.303	<0.001
Humor	2.55 (±1.18)	−0.117	<0.001	−0.015	0.532
Acceptance	4.92 (±1.68)	−0.028	0.233	−0.030	0.195
Religion	6.43 (±1.72)	0.239	<0.001	0.235	<0.001
Self-blame	3.97 (±1.76)	0.300	<0.001	0.435	<0.001
Self-distraction	4.83 (±1.64)	−0.042	0.072	0.096	<0.001
Denial	4.00 (±1.93)	0.186	<0.001	0.304	<0.001
Substance use	2.33 (±0.99)	−0.078	0.001	0.021	0.371
Behavioral disengagement	3.91 (±1.61)	0.234	<0.001	0.343	<0.001

SD, standard deviation.

### 3.5 Multivariate logistic regression for determinants of depression and anxiety

The multivariate logistic regression was used to explore the factors that predict the development of depression symptoms, as seen in Table 6. The findings showed that students from faculties of arts had significantly a higher probability of having depression compared to faculties of science and faculties of health (AOR: 1.399, *p*-value = 0.031). Also, living in the middle governorates had significantly a lower probability of having depression compared to the north governorates (AOR: 0.717, *p*-value = 0.020), and being a male student had significantly a lower odd of having depression compared to a female student (AOR: 0.713, *p*-value = 0.010). In addition, students whose fathers had a work during the political violence and wartime had a significantly lower probability of having depression compared to those who did not have work (AOR: 1.570, *p*-value = <0.001). Similarly, students whose mothers had worked during the political violence and wartime had significantly a lower probability of having depression compared to those who did not have work (AOR: 0.679, *p*-value = 0.001). Finally, using coping strategies including active coping (AOR, 0.823, *p*-value = 0.000), emotional support (AOR, 0.928, *p*-value =0.043), humor (AOR, 0.816, p-value = <0.001) reduced the probability of developing depression symptoms. However, using religion (AOR, 1.258, *p*-value = <0.001), self-blame (AOR, 1.283, p-value = <0.001), denial (AOR, 1.269, p-value = <0.001), and behavioral disengagement (AOR, 1.321, *p*-value = <0.001), increased the likelihood of having depression symptoms.

**Table 6:** Multivariate logistic regression to predict the determinants of the development of depressive symptoms.

Variables in the model	Significance	Adjusted odds ratio	95% C.I. for AOR	
			Lower limit	Upper limit
Facilities				
Faculties of Science	0.076	1.370	0.968	1.939
Faculties of Art (Humanities)	0.031	1.399	1.032	1.896
Facilities of Health	**Reference**			
Gender				
Male	0.010	0.713	0.552	0.922
Female	**Reference**			
Area of living				
Southern	0.077	1.266	0.975	1.644
Middle	0.020	0.717	0.541	0.949
Northern	**Reference**			
Father work				
Has work currently	0.001	0.679	0.545	0.845
No work	**Reference**			
Coping strategies				
Active coping	<0.001	0.823	0.760	0.892
Emotional support	0.043	0.928	0.863	0.998
Humor	<0.001	0.816	0.732	0.908
Religion	<0.001	1.258	1.175	1.347
Self-blame	<0.001	1.283	1.188	1.385
Denial	<0.001	1.269	1.151	1.400
Behavioral disengagement	<0.001	1.321	1.177	1.482

The logistic regression model was adjusted for: University, study year, faculty type, gender, living place, marital status, family income, area of living, father’s education, father’s work, mother’s education, mother’s work, age, active coping, use of informational support, positive reframing, planning, emotional support, venting, humor, acceptance, religion, self-blame, self-distraction, denial, substance use, and behavioral disengagement.

Moreover, multivariate logistic regression was used to explore the factors that predict the development of anxiety symptoms, as seen in Table 7. The findings showed that students from Al Quds University had significantly a lower probability of having anxiety symptoms compared to An-Najah University (AOR: 0.691, *p*-value = 0.006), and being a male student had significantly lower odds of having anxiety compared to female students (AOR: 0.521, *p*-value = <0.001). In addition, students whose fathers had worked during the political violence wartime had significantly a lower probability of having anxiety symptoms compared to those who did not have work (AOR: 0.784, *p*-value = 0.048). Additionally, using coping strategies including active coping (AOR: 0.90 7, *p*-value = 0.018), positive reframing (AOR: 0.928, *p*-value =0.036), humor (AOR: 0.855, *p*-value = 0.005), acceptance (AOR, 0.842, *p*-value = <0.001) decreased the probability of developing anxiety symptoms.

**Table 7:** Multivariate logistic regression to predict the determinants of the development of anxiety symptoms.

Variables in the model	Significance	Adjusted odds ratio	95% C.I. for AOR	
			Lower limit	Upper limit
University				
Al Quds University	0.006	0.691	0.530	0.902
Hebron University	0.578	1.087	0.810	1.459
An-Najah University	**Reference**			
Gender				
Male	0.000	0.521	0.397	0.684
Female	**Reference**			
Father’s work				
Currently working	0.048	0.784	0.616	0.998
No work	**Reference**			
Family monthly income ($)				
Less than 500	0.333	1.272	0.781	2.071
500 to 1,000	0.621	1.111	0.733	1.682
1,001 to 1,600	0.177	0.740	0.478	1.145
More than 1,600	0.240	0.764	0.488	1.197
No income	**Reference**			
Coping strategies				
Active coping	0.018	0.907	0.837	0.983
Positive reframing	0.036	0.928	0.865	0.995
Planning	0.002	1.156	1.054	1.268
Venting	0.003	1.149	1.048	1.259
Humor	0.005	0.855	0.767	0.952
Acceptance	0.000	0.842	0.777	0.912
Religion	0.001	1.146	1.061	1.238
Self-blame	0.000	1.492	1.370	1.625
Denial	0.018	1.088	1.014	1.167
Behavioral disengagement	0.000	1.247	1.146	1.358

The logistic regression model was adjusted for: university, study year, faculty, gender, living place, marital status, family income, area of living, father’s education, father’s work, mother’s education, mother’s work, age, active coping, \use of informational support, positive reframing, planning, emotional support, venting, humor, acceptance, religion, self-blame, self-distraction, denial, substance use, and behavioral disengagement.

*$, American dollars.

On other hand, using planning (AOR: 1.156, *p*-value = 0.002), venting (AOR, 1.149, *p*-value = 0.003), religion (AOR: 1.146, *p*-value = 0.001), self-blame (AOR, 1.492, *p*-value <0.001), denial (AOR: 1.088, *p*-value =0.018), and behavioral disengagement (AOR: 1.247, *p*-value <0.001), increased the likelihood of having anxiety.

## 4 Discussion

This study examined the prevalence of depression and anxiety among Palestinian university students during Israeli and Palestinian political violence and war after Octiber7, 2023. The results revealed a high prevalence of depression and anxiety. In addition, active coping, emotional support, and humor reduced depression symptoms, whereas active coping, positive reframing, humor, and acceptance reduced anxiety symptoms. Furthermore, religion, self-blame, denial, and behavioral disengagement increased the probability of having depressive symptoms, whereas planning, venting, religion, self-blame, denial, and behavioral disengagement increased the likelihood of experiencing anxiety.

The current study found that 65.9% of the participants had depression symptoms, whereas 60.9% had symptoms of anxiety. This finding is considered to be high in comparison to previous research. Lim et al.’s systematic review revealed that the prevalence of depression and anxiety significantly increased after conflict or war, with 28.9 and 30.7% of participants reporting depression and anxiety, respectively. Additionally, during the conflicts, 43.4% of people had anxiety, and 38.7% experienced depression (12). Furthermore, Hoppen et al.’s (33) systematic review conducted from 1989 to 2019 in countries affected by conflicts found that 23.31% of those who survived the war had high levels of depression (34). Chudzicka-Czupała et al. (11) found that 46.3% of Ukrainians had high anxiety during the conflict, while 46.5% reported experiencing high levels of depression. Furthermore, a study done by Al Saadi et al. (35) in Syria found that the prevalence of depression was 60.6% and anxiety was 35.1%. In contrast, other research showed a higher prevalence of depression and anxiety in comparison to the current study. For instance, a study done in Pakistan revealed that 75% of university students exhibited symptoms of depression, whereas 88.4% reported experiencing anxiety (19). Research done in Ukraine found that 97.8% of university students experienced a decline in their psycho-emotional well-being, with a significant proportion (84.3%) having symptoms associated with depression (36).

Palestinian undergraduate students are more likely to experience depression and anxiety symptoms, which could be attributed to the country’s history of political violence (35). The ongoing conflict between Israel and Palestine has a significant impact on the younger generation (35). Additionally, Palestinian university students face unemployment, poverty, insecurity, and siege (36). Furthermore, significant educational disruption during political violence and war times may raise undergraduate students’ risk of depression and anxiety [38.] All West Bank Palestinian universities have been closed since October 7 because of political violence. Zoom-based virtual education has replaced in-person teaching. In the first month after the political violence and new war, thousands of Palestinian students boycotted classes due to psychological distress caused by the deaths and injuries in Gaza. Lockdowns in West Bank cities, as well as remote learning, may have made students more vulnerable to social isolation and loneliness, exacerbating their depression and anxiety symptoms during the conflict. Ge et al. found a link between lonely living, isolation, inadequate relationships, and depression (37). Our study suggests that mental health service delivery and interventions should adapt to prevent difficulties with mobility and university or city closures during war or political violence. A computerized program can increase awareness of mental health issues among Palestinian undergraduates. Students can access the program via their university accounts at any time.

Moreover, our findings showed that arts or humanities students were more likely to experience depression than health and science students. Similarly, Banu et al. and Baviskar et al. reported that humanities or art students were more depressed than science students (38, 39). In contrast, Wani found that science students experienced higher levels of stress, anxiety, and depression (73.86, 96.14, and 88.46%) than art students (40). Health and science students may have a better knowledge of mental health issues like depression and anxiety, resulting in better emotional management. Our findings also revealed that middle governorate students were significantly less depressed than northern governorate students and Al Quds University students reported significantly lower levels of anxiety than students at An-Najah University in Nablus, Northern Palestine. This result could be due to an Israeli military operation in Jenin and Nablus in the northern governorate before the beginning of the political violence on October 7th. This political violence may have exacerbated the undergraduates’ depression and anxiety symptoms. Therefore, Palestinian universities should provide psychological interventions that target students from the northern governorate of Palestine.

Furthermore, male students in the current study were significantly less likely than female students to show signs of depression or anxiety. These findings are consistent with previous research, which found that female students are more stressed, anxious, and depressed than males (41, 42). Other research reported that men (67.35%) experienced more depressive symptoms than women (42). Some studies revealed no significant relationship between depression and gender, but a strong link between female gender and anxiety (43, 44). Additionally, our study found that students whose fathers worked during political violence had a lower risk of developing depression and anxiety symptoms compared to those who did not work. Similarly, students whose mothers worked during the war were significantly less likely to experience depressive symptoms than those who did not. Our findings are consistent with previous research, which found that low-income students are more likely to experience depression and stress (45–48). In contrast, Al Saadi et al. (41) found that while monthly family income had no significant effect on students’ psychological distress, attitude toward personal income was a significant predictor of depression and anxiety. Other research revealed no link between economic status and depression (49). The Palestinian economy has been experiencing a crisis since October 2023, including loss of life, financial damage, and unemployment. This has resulted in additional cuts and delays in public salary disbursements (50). Additionally, Palestinian workers were barred from entering Israel to work. Most Palestinian undergraduate students rely on family income as they have no other sources of income. Poverty has been consistently associated with poor mental health (51, 52). As a result, these factors may contribute significantly to the rise in mental health issues such as depression and anxiety symptoms among Palestinian undergraduate students.

These affective disorders could be caused by differences in how people deal with emotional distress. Our study found that active coping, emotional support, and humor reduced depression symptoms, while active coping, positive reframing, humor, and acceptance reduced anxiety symptoms. Problem-focused coping strategies including active coping and positive reframing are an active attempt to handle a stressful circumstance by participating in problem-solving activities to change the situation or seek alternatives. Positive emotional coping strategies, such as humor, and emotional support, involve showing compassion and empathy towards oneself while trying to solve a problem independently, regardless of the outcome. These strategies also involve making cognitive adjustments that help generate positive emotions and promote a sense of calmness in difficult situations (53). According to Stanisławski (53), acceptance is a positive emotion-focused coping strategy that is also linked to considerably reduced anxiety and stress scores. It involves the ability to accept the reality of a stressful situation (53). Our study supports the literature review’s findings that emotional support and active coping are effective at protecting undergraduate students in conflict zones from depression and anxiety (23, 54). Saxon et al. (25) found that humor, emotional support, active coping, and acceptance all contributed to better mental health in conflict zones. Garg et al. (55) indicated that emotional focused coping strategies such as humor, and emotional support reduce depressive symptoms. However, Mozid (56) revealed that emotion-focused coping strategies such as seeking emotional support, venting, humor, acceptance, self-blame, and religion had a significant negative correlation with psychological distress. Our study found a negative correlation between humor and depression and anxiety, supporting previous research (57). Stockton et al. (58) revealed that humor reduces depression, interpersonal suicide risk, suicidal ideation, and anxiety. Additionally, anxiety and acceptance were negatively correlated in our study, supporting the literature review (59, 60). Crișan et al. (59) found that adaptability-based acceptance is the most effective factor in reducing anxiety in political violence and war. Thus, students might get benefits from using these coping strategies to efficiently manage and modify the factors that contribute to their stress in the context of political violence and conflict. Furthermore, students can manage and control their emotional responses to these difficult situations, perhaps leading to a reduction in symptoms associated with depression and anxiety.

Additionally, the current study found that religion, self-blame, denial, and behavioral disengagement increased the likelihood of experiencing depression symptoms while planning, venting, religion, self-blame, denial, and behavioral disengagement increased the likelihood of experiencing anxiety. Similarly, Awoke et al. reported that using denial, self-blame, planning, and religion to cope with stress was associated with high perceived stress among undergraduate students (61). Crișan et al. (59) showed that venting emotions and behavioral disengagement had the greatest negative impact on anxiety and well-being. Furthermore, Thapa et al. (62) study found that coping strategies like denial, positive reframing, and religion had a negative relationship with stress in university students. Furthermore, Garg et al. (55) reported that avoidant coping mechanisms (self-distraction, denial, and behavioral disengagement), and emotional focused coping strategy (venting) were significantly associated with increased depressive symptoms in undergraduate students. Additionally, the Saxon study found that coping strategies such as behavioral and mental disengagement, denial, emotional venting, substance abuse, and gambling were significantly associated with poorer mental health outcomes (25). According to Deng et al. (46) undergraduate students are unable to self-manage their emotions and have not developed coping strategies for stressful experiences, making them vulnerable to depression and anxiety. These findings suggest the significance of creating an intervention program focused on teaching effective coping strategies to undergraduate students during periods of political violence and war.

This research had some limitations. Using convenience sampling limits the generalizability of our findings to a wider population of Palestinian university students. Also, the study’s cross-sectional design limits causal inferences about the relationship between exposure to political violence and mental health outcomes. Additionally, using self-reported questionnaire increases the possibility of reporting bias. Further, this study only included three Palestinian universities, which may not reflect the diversity of the student population in Palestine. In addition, students at other universities may experience different levels of anxiety and depression than those who participated in this study. Despite these limitations, the results shed new light on the mental health of undergraduates in areas with ongoing conflicts, and those known to suffer from a disproportionately high rate of mental diseases like depression and anxiety. This study adds significantly to the existing literature by being the first of its kind to examine the symptoms of anxiety and depression as well as coping mechanisms among Palestinian undergraduates who were enrolled during periods of political violence and conflict.

### 4.1 Implication of the study and future research

The study suggests screening and intervention programs to prevent and treat mental health problems among undergraduate students studying in conflict zones. Our findings highlight the need to use an innovative approach to delivering mental health treatments and interventions to address difficulties such as restricted mobility and dealing with the closure of universities or cities during times of war or political instability. Implementing a computerized program might be an effective strategy to enhance knowledge about the symptoms of mental or psychological problems that Palestinian undergraduate students may experience. Students can access this program at their leisure by logging into their university accounts. Furthermore, mental health practitioners and university officials should offer clinical and educational interventions to help students develop effective coping strategies and enhance their emotional well-being in the face of political violence.

More studies are needed to determine the psychological impact of political violence on the well-being of students in communities and families during and after the war. Future research is needed to investigate additional risk factors for depression and anxiety, such as fear of war, stress, and feelings of loneliness.

## 5 Conclusion

The study found that political violence often leads to symptoms of depression and anxiety among undergraduates. Furthermore, the use of maladaptive coping mechanisms increases the likelihood that these symptoms will occur. Providing immediate assistance to university students affected by political violence and conflicts is crucial for their emotional and mental recovery and coping with difficulties.

## Data Availability

The original contributions presented in the study are included in the article/supplementary material, further inquiries can be directed to the corresponding author/s.
